# Effects of Trained Peer vs. Teacher Feedback on EFL Students’ Writing Performance, Self-Efficacy, and Internalization of Motivation

**DOI:** 10.3389/fpsyg.2021.788474

**Published:** 2021-11-24

**Authors:** Ying Cui, Christian D. Schunn, Xiaosong Gai, Ying Jiang, Zhe Wang

**Affiliations:** ^1^School of Psychology, Northeast Normal University, Changchun, China; ^2^Learning Research and Development Center, University of Pittsburgh, Pittsburgh, PA, United States; ^3^Institute of Education and Science, Jilin Engineering Normal University, Changchun, China

**Keywords:** teacher feedback, peer feedback, training, writing ability, writing motivation

## Abstract

This study investigated the longer-term impacts (i.e., into the next semester) of trained peer feedback in comparison with teacher feedback on students’ writing development and writing motivation. Sections of an EFL writing course were randomly assigned to either teacher feedback or trained peer feedback conditions across two semesters. In the first semester, during their writing class, students either received training in how to implement peer feedback or simply studied models of writing (that were also used in the training work). In the second semester, students either received teacher or peer feedback across multiple assignments. Writing competence, writing self-efficacy, and writing self-regulated learning were assessed at the beginning and end of the second semester. Trained peer feedback and teacher feedback had similar positive effects on the improvement of writing competence and writing self-efficacy. However, trained peer feedback led to a significant enhancement of students’ autonomous motivation relative to no such growth from teacher feedback.

## Introduction

Learning to write in English is a major learning challenge around the world (Li, 2020; Tao, 2020), and the availability of frequent, timely, and accurate feedback is central to that challenge (Ferris and Roberts, 2001; Hyland and Hyland, 2006; Lee et al., 2021). A great deal of research has already examined the effects of peer and teacher feedback on ESL and EFL students’ revision quality and writing performance (Fathman and Whalley, 1990; Leki, 1990; Connor and Asenavage, 1994; de Guerrero and Villamil, 1994; Ferris, 1997; Villamil and Guerrero, 1998; Berg, 1999; Paulus, 1999; Tsui and Ng, 2000; Saito and Fujita, 2004; Rollinson, 2005; Min, 2006; Yang et al., 2006; Zhang and Dai, 2011; Ruegg, 2015; Cui et al., 2019, 2021; Zhang and Cheng, 2020), especially when peers have received some training in how to give effective feedback (Berg, 1999; Min, 2006). And there is also some research investigating the effects of peer feedback and teacher feedback on ESL and EFL students’ writing self-efficacy (Chaudron, 1984; Tsui and Ng, 2000; Ruegg, 2018; Fathi et al., 2019; Lee and Evans, 2019), which is also important because self-efficacy of writing plays an important role in predicting students’ future writing performance (Zimmerman and Bandura, 1994; Pajares and Johnson, 1996; Klassen, 2002; Woodrow, 2011). However, feedback on writing can have other effects that are important to long-term effects (i.e., beyond the specific course) on students’ writing ability such as improving autonomous motivation (McMahon, 2010; Yousefifard and Fathi, 2021), a critical goal of foundation courses. Here, we test the relative benefits of teacher vs. trained peer feedback in an EFL context on writing ability, self-efficacy, and autonomous motivation.

### Peer Feedback’s Role in the Development of Writing Ability

A number of recent meta-analyses have firmly established the value of peer feedback in learning to write (e.g., Huisman et al., 2019; Zheng et al., 2019). Vygotsky (1978) argued that social interaction is a critical component of cognitive learning, and peer feedback is a kind of pedagogical activity that is often connected to Vygotsky. For example, Villamil and Guerrero’s (1998) study of peer feedback in interactive groups based on Vygotsky’s theoretical framework found that scaffolding occurred in the interaction process of peer feedback group, making students in the group learn from each other, and the learning results not only include rhetorical, content and other aspects, but also include the overall development of language.

In the process of cognitively and behaviorally engaging with peer feedback (Fan and Xu, 2020), students improve their writing ability by improving their writing awareness. For example, Tsui and Ng (2000) found that students can improve their understanding of the advantages and disadvantages of their own writing by using peer feedback. Improving students’ understanding of writing modification strategies is an important part of the development of second language writing skills (Hedgcock and Lefkowitz, 1992). Berg’s (1999) study of peer feedback in writing activities found that when students exchanged opinions from each other and respond to trained peer feedback, it produced a foundation that helped students complete writing tasks in the future.

### Writing Self-Efficacy

In addition to actual ability, there is self-efficacy, the self-perception of one’s ability (Bandura, 1986). Self-efficacy is important to learning because the more self-efficacy students have, the more they will persevere in their efforts (Bandura, 1989). Self-efficacy has been identified as a key factor to success in language learning (Bown and White, 2010; Prat-Sala and Redford, 2010). Writing self-efficacy, confidence in one’s own writing ability, has been found to predict student’s writing achievement (Zimmerman and Bandura, 1994; Pajares and Johnson, 1996; Klassen, 2002; Woodrow, 2011; Han and Hiver, 2018).

Theoretically speaking, engaging with peer feedback gives students extensive feedback, and sometimes feedback that is less harsh than teacher feedback. Thus, peer feedback could improve student self-efficacy in a number of ways. Further, other forms of peer interaction have been found to improve self-efficacy (e.g., Rahimi and Fathi, 2021; Shin and Johnson, 2021). However, studies on the effect of peer feedback on writing self-efficacy have obtained mixed results. Some studies found that peer feedback had a positive effect on students’ writing self-efficacy (Chaudron, 1984; Tsui and Ng, 2000; Lee and Evans, 2019). By contrast, Ruegg (2018) found that peer feedback produced no improvement in students’ writing self-efficacy, and even showed a decrease in grammar self-efficacy. Training students in how to give good peer feedback may be needed to obtain motivational benefits. It is also unclear to what extent the value is peer feedback is for writing self-efficacy or feedback (on writing) self-efficacy, the more specific thing being practiced. No previous research has examined peer feedback’s effect on different relevant domains of self-efficacy.

### Internalization of Motivation

Another motivational framework that is especially relevant to peer feedback is Deci and Ryan’s Self Determination Theory (SDT; Deci and Ryan, 1985). This theory of motivation assumes three fundamental needs: competence, relevance, and autonomy. Across studies in many different domains (Williams and Deci, 1996; Black and Deci, 2000; Standage et al., 2003), the satisfaction of these basic psychological needs leads to improved motivation to complete tasks (including learning tasks) and general psychological well-being. Competence closely matches Bandura’s concept of self-efficacy, which was discussed in the prior section. Of the remaining two components of SDT, autonomy is particularly relevant to peer feedback.

According to Ryan and Deci (2000), in pursuing goals, a learner can do so in autonomously (self-directed) or in controlled (other-directed) fashion, and the learner will be more satisfied if they do so autonomously. Peer feedback could improve student’s sense of autonomy in that they practice error detection and revising while providing feedback, which are critical skills that they could apply to their own writing without always depending upon teacher feedback (Yang et al., 2006; Shen et al., 2020).

No prior work has specifically examined peer feedback effects on autonomous vs. controlled motivation. On the one hand, the peer feedback *received* may be experienced as just another kind of controlled motivation. On the other hand, *providing* feedback to others might lead to having greater autonomy in later writing. In another collaborative learning context, peer interaction in a discussion board, students showed increases in their autonomous motivation through feedback received from other students (e.g., Xie and Ke, 2011; Xie, 2013). However, sometimes autonomous motivation effects are dependent upon feeling sufficiently competent (Shin and Johnson, 2021). We focus on the case of EFL writing, where students often have very low self-efficacy. In such a case training in how to give effective feedback may be needed in order to see growth in autonomous motivation from engaging in peer feedback activities.

### Purposes and Research Questions of the Present Study

Despite the existence of many studies on peer vs. teacher feedback, no studies have systematically compared them in terms of their relative effects on writing performance, self-efficacy, and internalization of motivation. In addition, some studies in ESL and EFL contexts in particular have suggested that peer feedback can improve both the quantity and quality of text revisions after students are trained to provided peer feedback (Stanley, 1992; Berg, 1999; Min, 2005, 2006; Lam, 2010), but these studies have not systematically examined the training benefits across motivational domains.

This study addressed three research questions related to relative effects of teacher feedback vs. trained peer feedback:

1. What are relative effects on students’ writing ability?
2. What are relative effects on different aspects of students’ writing self-efficacy?
3. What are relative effects on students’ autonomous and controlled motivation?

## Materials and Methods

### Participants

The 122 participants were a convenience sample of all enrollees in a writing course (described below). They were English majors (111 women; 11 men) who were third year undergraduate students (mean age of 21) at a private university in northeastern China. All spoke Mandarin as their first language and had received formal English training for more than 8 years at the time of the study. However, their average score on the Test for English Majors-Band 4 (TEM-4) was only 50 out of 100, which is a relatively low score. They had not previously received training on peer feedback before the study. Twenty-eight participants were excluded because they failed to submit papers or questionnaires, leaving 94 in the study.

### Course Setting and Research Design

This study was conducted within four sections of two semester-long course, *Intermediate English Writing*, offered for English majors. There were 33, 32, 32, and 25 students in the four sections, respectively. The main objective of the course is to develop writing skills in argumentation, but there was also continued work to improve more fundamental aspects of English. The instructor, who was also one of the research team members, taught all four sections and met the students once a week for 19 weeks in the first semester and 17 in the second semester, with each class session lasting 90 min. Students wrote three formal out-of-class papers during the first semester, all with teacher feedback, while some students were receiving training on giving feedback. In the second semester, there were four out-of-class papers. Each paper writing assignment involving a first draft completed as homework, feedback provided by either teacher or peer and finally a second draft completed as homework.

The research design was a between-groups design involving two conditions. The *Trained Peer Feedback* group involve two sections (48 students), and the *Teacher Feedback* group involved the other two sections (46 students). As show in Table 1, students in both conditions practiced writing with only teacher feedback in the first semester. The difference between the two groups in the first semester is that students in the Trained Peer Feedback group received training on how to give feedback (described below), whereas the students in the Teacher Feedback group received similar materials (e.g., model essays used in the training) but did not receive the training on peer feedback. In the second semester, no additional “extra” training was provided beyond the traditional writing instruction that was common the two conditions. Instead, the difference between conditions in the second semester is that the Trained Peer Feedback group received only peer feedback for the four assigned papers and the Teacher Feedback group received only teacher feedback for the four assigned papers. For the purposes of the study, the first semester is treated as the training, and the second semester is the main focus for measuring impact of training on students’ writing improvement and attitudinal changes. In other words, what were the relevant benefits of peer vs. teacher feedback in a situation in which peers had been previously trained on how to give peer feedback?

**TABLE 1:** Overview of writing and training across conditions: training in the first semester, contrast of peer vs. teacher feedback in the second semester (key condition differences in bold).

Semester	Trained Peer Feedback group		Teacher Feedback group	
	Writing work	Extra training	Writing work	Extra training
First	Three papers with teacher feedback	**Feedback training** on model essays	Three papers with teacher feedback	**Examination** of model essays
Second	Four papers with **peer** feedback	NA	Four papers with **teacher** feedback	NA

Throughout both semesters, class instruction (and corresponding textbook units) around the time of each writing assignment emphasized a particular aspect of argumentation (i.e., organization, claim, refutation, or emotional appeal) and a particular component of argument writing (outline, introduction, body, conclusion). The specific writing assignments in the focal (second) semester were taken from the textbook and involved argumentative writing on topics of general interest to university students: “Campus Love – Pros or Cons” (Topic 1), “Icon Worship” (Topic 2), “Examinations: For or Against” (Topic 3), and “Beauty, from Heart or Face? (Topic 4).” However, the basic writing task was the same across all four assignments and the same overall evaluation rubric was applied to each document.

The order of the first and last writing task topics as writing prompts was varied across the two sections within each group. This counterbalancing of writing topics allows for more precise measurement of writing improvement while controlling effects of writing prompt on student performance. The order of writing topics in each of the four course sections is shown in Table 2.

**TABLE 2:** Order of writing topics in each course section/condition during the second semester.

Course section	Order of second semester writing assignments			
Teacher feedback 1	Topic 1	Topic 3	Topic 4	Topic 2
Teacher feedback 2	Topic 2	Topic 3	Topic 4	Topic 1
Trained peer feedback 1	Topic 1	Topic 3	Topic 4	Topic 2
Trained peer feedback 2	Topic 2	Topic 3	Topic 4	Topic 1

### Measures

#### Writing Quality

To study condition effects on writing quality improvements from feedback, first drafts of the first and fourth writing assignments in the second semester were expert scored in order to measure the gains across the semester in writing performance. The drafts were scored by two experts (English teachers) who were blind to condition and draft number, using rubrics from the TEM-4, involving: aspects of meaning, organization, and surface issues. Both graders had extensive prior experience using these rubrics. The inter-rater correlation in overall draft score was high, *r* = 0.74, *p* < 0.01.

#### Writing Self-Efficacy

A writing self-efficacy questionnaire was adapted from the Self-efficacy Scale of College Students’ English Writing compiled by Li (2014). It included questions from the original questionnaire about writing skill (nine questions) and writing task efficacy (nine questions). To add an assessment of self-efficacy for providing feedback on writing, 13 questions were added (see Table 3 for examples of each self-efficacy dimension). The questions used a 0–100 scaled based upon findings from Pajares et al. (2001) that this scale worked well for investigating writing self-efficacy. Scale reliability in the current study sample was acceptably high for each dimension (see Table 3).

**TABLE 3:** Survey measures, scale reliabilities, number of items, and example items.

Measure (and Cronbach α)	Number of items	Example item (English translations)
Writing self-efficacy (0.97)	31	
Skill (0.87)	9	I can accurately use singular and plural in English writing.
Task (0.92)	9	I can write a convincing argument to express myself effectively in English.
Feedback (0.95)	13	I can find mistakes about grammar in English writing.
Writing self-regulated learning	12	
Autonomous (0.75)	5	I actively participate in writing classes because it is a good way to improve my writing skills.
Controlled (0.77)	7	If I don’t actively participate in the class, others will think I am a poor student.

#### Writing Self-Regulated Learning

A writing self-regulated learning questionnaire was adapted from the Black and Deci (2000) survey based upon self-determination theory. The questionnaire focused on why people take college writing courses and consisted of two dimensions: autonomous motivation (i.e., five questions related to identity regulation or internal motivation) and controlled motivation (i.e., seven questions related to external regulation or introjected regulation). A traditional 7-point Likert scale was used. Table 3 shows example questions and Cronbach α for each scale in the current study sample; the two scales showed adequate reliability.

### Procedure

#### Peer Feedback Training

The extra training provided to the Trained Peer Feedback group was distributed across writing units in the first semester, applying a training model built upon emerging best practices of prior writing teachers (Berg, 1999; Min, 2005, 2006; Lam, 2010). The main training was divided into several parts that were repeated across course topic units: small group discussion, teacher answering student questions, and teacher modeling.

Students created their own groups of 3–4 students for the small group discussion. They completed a task in class (e.g., writing an outline and writing an introduction), and gave each other oral feedback in their groups. While students were working in their small groups, the teacher would circulate and answer student questions. In particular, the students commonly had questions about whether an issue they noticed was actually a problem, which of multiple potential solutions was better, or competing opinions about a document. The purpose of this step was to model strategies for resolving ambiguities in feedback giving.

Then the teacher randomly selected several students’ writing work (e.g., outlines) as examples to demonstrate how to give feedback and make suggestions for revision. In particular, the modeling emphasized how to give feedback on both language and content aspects of writing. This approach was repeated as students worked on each part of argumentation (introduction, body, and conclusion). Toward the end of the semester, as students practiced writing complete argumentative essays, the teacher chose five model argumentative essays to demonstrate how to give feedback. The modeling involved a Peer Feedback Form (Appendix I) that peers would then use for their peer reviewing. In particular, the instructor explained the items on the guidance sheet with illustrations.

Given the EFL context, additional training was included for how to give useful feedback on surface level writing issues. Specifically, the training emphasized using a dictionary to determine the meaning of words and using the Internet to find relevant grammar knowledge.

#### Teacher Feedback Condition Supplementary Training

In the Teacher Feedback conditions, in place of the peer feedback training in the first semester, the teacher provided a corresponding good writing example and explained why the writing example was effective. Because the topics of instruction shifted, these explanations also shift in their focus from outlining, introduction, and so on.

#### Teacher and Peer Feedback

In the second semester, students received either teacher or peer feedback on each of the four writing assignments. The same feedback form was used in both conditions. To avoid potential bias, the teacher feedback was from another teacher in the same department who was paid for this additional work. The teacher was asked to use the feedback form, and otherwise consistently follow the same feedback practice that they had used as a teacher in prior years of teaching this course. Peer feedback was given to each other in small groups of 3–4 students within the same class. Both peers and the teacher were asked to look for instances of plagiarism because plagiarism tends to disrupt authentic conversations about student writing.

#### Data Collection Procedures

During the semester, students in both conditions completed questionnaires about writing self-efficacy and writing self-regulated learning as pre-test (during the first week of class) and post-test (during the last week of class). The pre-test contained demographic questions about age and gender. The questionnaires were completed on paper and non-anonymously so that pre and post tests could be linked during analysis. Students’ writing were submitted electronically by email to the instructor as well as printed to be shared with peers.

### Analyses

The data was screened for outliers. Outlier values were found for one participant on self-efficacy ratings and were winsorized (i.e., values were replaced by the 2 SD threshold values). Homogeneity of variance and normality assumes were checked using Levine’s tests and the Shapiro–Wilk, respectively, and the assumptions were found to be generally met. However, the distributions for post self-efficacy and pre and post autonomous motivation had highly skewed distributions. Transforming those variables into binary variables (above the mode or not) and then reanalyzing using those transformed variables produced identical results. For simplicity, the untransformed results are presented here.

Initial condition differences at pre-test are examined using unpaired *t*-tests and Cohen’s *d*.

To test the main research questions, a mixed between-within ANOVA was used to examine pre–post changes, and interactions between condition and pre–post change for overall writing competence, writing self-efficacy and writing motivation. η^2^ was used to characterize effect sizes of main effects and interactions. *t*-Tests and Cohen’s *d* were used to calculate the statistical significance and effect sizes of pre–post changes within each condition.

## Results

Pre and post means, along with effect size and statistical significance of pre–post changes on each measure are presented in Table 1. We examine patterns of change within each outcome variable in the following sections (see Table 4).

**TABLE 4:** Mean (and SD) pre/post values and Cohen’s *d* of the pre–post growth in each condition for writing competence, writing self-efficacy, and self-regulated motivations.

	Teacher feedback			Trained peer feedback		
	Pre	Post	Pre–post *d*	Pre	Post	Pre–post *d*
Writing competence	12.1 (1.1)	12.8 (1.2)	0.58**	12.6 (1.2)	13.0 (1.1)	0.39*
Writing self-efficacy						
General	66% (11%)	73% (9%)	0.72**	74% (13%)	82% (8%)	0.70**
Skill	72% (10%)	77% (10%)	0.49**	78% (13%)	81% (10%)	0.25*
Task	64% (12%)	72% (10%)	0.74**	73% (12%)	78% (9%)	0.44**
Feedback	62% (13%)	70% (12%)	0.63**	72% (15%)	85% (6%)	1.08**
Motivation						
Autonomous	29.4 (3.6)	29.7 (3.3)	0.08	29.3 (3.6)	31.8 (2.1)	0.85**
Controlled	31.8 (6.7)	32.1 (6.7)	0.04	30.6 (6.8)	33.4 (6.9)	0.41

**p < 0.01, *p < 0.05.

### Initial Condition Differences

At the beginning of the second semester, the Trained Peer Feedback group was higher on writing competence with moderate effect size (*t* = 2.08, *p* = 0.05, *d* = −0.42). Similarly, there were also moderately sized initial differences in overall writing self-efficacy (*t* = 3.56, *p* < 0.01, *d* = −0.74), skill self-efficacy (*t* = 2.45, *p* < 0.05, *d* = −0.51), task self-efficacy (*t* = 3.72, *p* < 0.01, *d* = −0.77), and feedback self-efficacy (*t* = 3.42, *p* < 0.0.01, *d* = −0.71). There were not meaningful initial differences in either autonomous motivation (*t* = 0.08, *p* > 0.05, *d* = 0.02) or controlled motivation (*t* = 0.87, *p* > 0.05, *d* = 0.18). These initial differences are not the main focus of this experimental design, which formally analyzes differences in pre–post growth. Further, these differences could reflect by-chance variation in section enrollment patterns. However, these differences may also reflect initial benefits of the peer feedback training procedures, which should be more formally investigated in future research.

### Changes in Writing Competence

Students in both conditions showed moderate pre–post gains in writing competences [*F*(1,92) = 18.00, *p* < 0.01, η^2^ = 0.16]. The slightly large gains in the Teacher Feedback group were not statistically significant (*F* < 1, η^2^ = 0.01). Thus, Trained Peer Feedback group was not disadvantaged despite receiving no teacher feedback on writing throughout the second semester.

### Changes in Writing Self-Efficacy

Students in both conditions showed large gains from pre to post-test in overall writing self-efficacy [*F*(1,92) = 67.38, *p* < 0.01, η^2^ = 0.42], and there was no interaction in the amount of pre–post gain between the two groups (*F* ≪ 1, η^2^ = 0). If the initial differences in self-efficacy were a result of the training process, these relatively gains were maintained through the second semester experiences.

There were overall pre–post gains in each more specific self-efficacy type, with smaller overall gains in skill self-efficacy [*F*(1,92) = 16.71, *p* < 0.01, η^2^ = 0.15], moderate overall gains in task self-efficacy [*F*(1,92) = 36.09, *p* < 0.01, η^2^ = 0.28], and large overall gains in feedback self-efficacy [*F*(1,92) = 89.39, *p* < 0.01, η^2^ = 0.49]. The relative pre–post gains were not statistically significant from one another by condition for skill self-efficacy (*F* = 1.16, *p* > 0.05, η^2^ = 0.01) or task self-efficacy (*F* = 2.43, *p* > 0.05, η^2^ = 0.03). However, the Trained Peer Feedback group showed larger gains in feedback self-efficacy (*F* = 3.73, *p* = 0.056, η^2^ = 0.04). Thus, switching from teacher to peer feedback did not disrupt general writing self-efficacy, and it promoted feedback self-efficacy.

### Changes in Self-Regulated Learning

**FIGURE 1:**
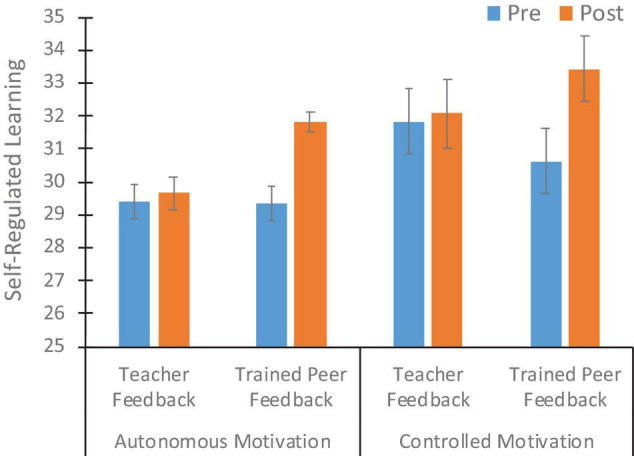
Mean (with SE bars) pretest and posttest self-regulated learning scores by condition.

Figure 1 presents the pre–post changes in self-regulated learning in each condition. There were small and statistically significant gains across conditions in autonomous motivation [*F*(1,92) = 4.74, *p* < 0.05, η^2^ = 0.05] and non-significant overall gains in controlled motivation [*F*(1, 92) = 2.02, *p* > 0.05, η^2^ = 0.02]. However, the gains were highly localized to the Trained Peer Feedback group. The interaction between gain and group was substantial and statistically significant for autonomous motivation (*F* = 6.05, *p* < 0.05, η^2^ = 0.06). As shown in both Figure 1 and Table 2, the Trained Peer Feedback group showed a large gain in autonomous motivation, whereas the Teacher Feedback group showed essentially no pre–post changes. Although showing a similar pattern of gains in only the Trained Peer Feedback group, the controlled motivation had higher variability in scores, and the interaction was not statistically significant (*F* = 1.44, *p* > 0.05, η^2^ = 0.02).

## Discussion

This study sought to investigate the longer-term impact of a scalable peer feedback training on revision in EFL writing classes. Impacts were examined on writing improvement and a number of motivational constructs. The training method requires only in-class work for teacher and students over a few class periods and is therefore one that can be easily integrated into the writing teaching curriculum, including when teachers have larger numbers of learners to support (e.g., over 120 students in a semester, as in the current study). Note that the focus here is not of the immediate effects of the training but rather on its delayed impact through the higher quality peer feedback that results.

The findings also deepen prior research on the effects of peer feedback training by examining both effects on writing competence and learner motivation. In recent years, several meta-analyses have established the benefits of peer feedback on learner’s writing/task performance (Double et al., 2019; Huisman et al., 2019), including in ESL and EFL classrooms (Thirakunkovit and Chamcharatsri, 2019). Prior findings in both ESL and EFL writing contexts have shown that peer feedback leads to improvements in self-efficacy (Chaudron, 1984; Tsui and Ng, 2000; Ruegg, 2018; Lee and Evans, 2019), similar to benefits of related pedagogical techniques like student blogging (Yousefifard and Fathi, 2021) and wiki writing (Rahimi and Fathi, 2021). Such benefits of peer feedback are likely the result of the combined effect of the training on peer feedback and the opportunities to practice peer feedback. Although the impact of peer feedback training was not independently studied and measured in this study, but it can be seen from the experimental results that after the training (in the second semester), the students in the peer feedback group had significantly higher initial writing scores and self-efficacy than those in the Teacher Feedback group. Past research had already established that engaging in peer feedback improves student’s self-efficacy; the current study extends that research to show that training in peer feedback immediately improves student’s self-efficacy but then also has delayed benefits in improving self-efficacy further as students engage in applying what they had learned during training.

Another novel contribution of the current study involves the specific aspects of self-efficacy that increased in response to peer and teacher feedback. All forms of writing self-efficacy showed improvements from pre to post in both feedback conditions. However, teacher feedback produced larger gains on self-efficacy for the writing task itself and underlying writing skills, whereas peer feedback produced larger gains on feedback self-efficacy (i.e., the exact task being practiced by engaging in peer feedback). It should be noted that the teacher feedback condition appeared to have a bit of a “catch-up” effect in that students in the trained peer feedback condition appeared to start ahead of students in the other condition in terms of skill and task self-efficacy. This early gains pattern may reflect deeper exposure to writing evaluation dimensions as part of training on peer feedback.

As a particularly novel finding, the current study revealed that trained peer feedback can significantly promote students’ autonomous motivation. From a self-regulated learning perspective, such motivational changes seem particularly important, especially in EFL contexts where students are very focused upon/dependent upon teacher feedback. Autonomous motivation is both relevant to self-regulated in later writing situations as well as supporting growth in intrinsic motivation for writing (Shen et al., 2020). It is worth noting that after the semester of training, writing competence and writing self-efficacy were significantly higher, but autonomous motivation did not differ between the two groups, which suggests that students’ improvements in autonomous motivation comes from the actual application of peer feedback.

Overall, at a theoretical level, the current study has drawn attention to the importance and separable effects of training on feedback for initial effects vs. later impacts of engaging in peer feedback. Peer feedback in general is meant to have long-term effects on students, both by improving their motivation and self-regulated learning, but also by providing a kind of writing process that will lead to future benefits (i.e., seeking out and willing participating in peer feedback).

At a pragmatic level, the current study tested a particular approach to peer feedback that can be deployed in situations where instructors have a large teaching load. Some previously tested approaches were relatively labor intensive in that teacher’s gave individual feedback on each student’s peer feedback (e.g., Min, 2006; Lam, 2010), which requires extensive out-of-class work for an instructor with many students. In the current approach, only class time is consumed for the training processes.

### Caveats and Future Research

The current study used a systematic experimental design with a carefully structured control condition and measurements at multiple time points using validated instruments. However, the generalizability of any such an experimental study needs to be tested, including in different learning contexts, with different writing genres, with different teachers doing the training, and using differential procedures and tools for supporting peer feedback. The instruments used in the current study seemed to function well and are therefore recommended for use in such later studies.

Secondly, while extending the results to later time points that is normally considered in training studies, future research should examine even longer time scales. Peer feedback can be used in any course or project context, and it remains unknown how long benefits of trained peer feedback and of training on peer feedback extend. For example, it is possible that students begin to forget the lessons about best practices in peer feedback or that the self-efficacy and autonomous motivation benefits wane as more difficult writing tasks are attempted.

## Conclusion

We draw three main conclusions from this study. Firstly, the application of post-training peer feedback in an English class can significantly improve students’ writing ability and writing self-efficacy. At the same time, there is no significant difference in the improvement of students’ writing ability and writing self-efficacy after using peer feedback compared with those who receive teacher feedback. Secondly, as a result of the first point, the very labor-intensive teacher feedback can therefore be safely reduced and replaced by more scalable peer feedback. Thirdly, the use of peer feedback can promote the improvement of students’ autonomous motivation in writing, which is likely to have many benefits in future writing contexts. The current research shows that it is feasible to apply peer feedback to students with relatively weak English proficiency, and it has a positive effect on the development of students’ writing. At the same time, simple and scalable models for training students before they use peer feedback are presented and validated here.

## Data Availability Statement

The original contributions presented in the study are included in the article/supplementary material, further inquiries can be directed to the corresponding author.

## Ethics Statement

Ethical review and approval was not required for the study on human participants in accordance with the local legislation and institutional requirements. Written informed consent for participation was not required for this study in accordance with the national legislation and the institutional requirements.

## Author Contributions

YC was the one who design the experiment, instructor, and data collector of the experiment. XG was supervisor of YC, gave guidance design of experiment, data analysis, and provided experimental funds. CS gave guidance on the writing and data analysis. YJ and ZW helped on data collection and analysis. All authors contributed to the article and approved the submitted version.

## Conflict of Interest

The authors declare that the research was conducted in the absence of any commercial or financial relationships that could be construed as a potential conflict of interest.

## Publisher’s Note

All claims expressed in this article are solely those of the authors and do not necessarily represent those of their affiliated organizations, or those of the publisher, the editors and the reviewers. Any product that may be evaluated in this article, or claim that may be made by its manufacturer, is not guaranteed or endorsed by the publisher.

## Appendix I

PEER REVIEW SHEET

Draft written by _________________________________________

Review written by _______________________________________

Date________ Composition___________________

Please answer the following questions with the purpose to provide your classmate with honest but helpful reactions and responses as the reader of this essay.

1. What do you like the best about the ideas in this essay? Be specific. (Precise vocabulary, cohesive/linked ideas, clear/easy to follow, convincing, effective reasoning, well-developed ideas, attention-grabbing introduction, strong conclusion, intriguing style, well supported topic sentences, understandable transitions, etc.)
2. Can you find a thesis statement? Can you find a clear topic sentence in each body paragraph? If you can, in your own words, state the focus/thesis/topic of the writing.
3. How many reasons and supporting proof are provided? Do all of these reasons logically support the writer’s opinion? Explain how well do these reasons persuade you that the author’s opinion is the correct one?
4. Are there any ideas in the essay that are not clear or that you find confusing?Mark these with a letter C. Explain why you think this is confusing and make some suggestions for improvement.
5. Are there any ideas in the essay that need further development? About which parts of the essay would you like more information? Mark these with a letter D. Explain why you think this should be developed more and make some suggestions.
6. How effective is the conclusion?
7. What questions, comments, and/or suggestions do you have for the author?
