# Corrigendum: Effects of Trained Peer vs. Teacher Feedback on EFL Students' Writing Performance, Self-Efficacy, and Internalization of Motivation

**DOI:** 10.3389/fpsyg.2022.878147

**Published:** 2022-03-22

**Authors:** Ying Cui, Christian D. Schunn, Xiaosong Gai, Ying Jiang, Zhe Wang

**Affiliations:** ^1^School of Psychology, Northeast Normal University, Changchun, China; ^2^Learning Research and Development Center, University of Pittsburgh, Pittsburgh, PA, United States; ^3^Institute of Education and Science, Jilin Engineering Normal University, Changchun, China

**Keywords:** teacher feedback, peer feedback, training, writing ability, writing motivation

In the original article, the reference for “Cui, Y., Schunn, C. D., and Gai, X. (2021)” was incorrectly written as “Cui, Y., Schunn, C. D., and Gai, X. (2021). Peer Feedback and Teacher Feedback: A Comparative Study of Revision Effectiveness in Writing Instruction for EFL learners. UK: Routledge. doi: 10.1080/07294360.2021.1969541” Instead, it should be written as “Cui, Y., Schunn, C. D., & Gai, X. (2021). Peer feedback and teacher feedback: a comparative study of revision effectiveness in writing instruction for EFL learners. Higher Education Research & Development. doi: 10.1080/07294360.2021.1969541.”

Additionally, in the original article, there was an error in the text. A full stop was missing, which altered the meaning of the text.

A correction has been made to **Introduction**, **Internalization of Motivation**, Paragraph 2. The corrected paragraph is shown below.

According to Ryan and Deci (2000), in pursuing goals, a learner can do so in autonomously (self-directed) or in controlled (other-directed) fashion, and the learner will be more satisfied if they do so autonomously. Peer feedback could improve student's sense of autonomy in that they practice error detection and revising while providing feedback, which are critical skills that they could apply to their own writing without always depending upon teacher feedback (Yang et al., 2006; Shen et al., 2020).

There was also an error in the **Materials and Methods** section. The average score on the Test for English Majors-Band 4 (TEM-4) was stated incorrectly.

A correction has been made to **Materials and Methods**, **Participants**, Paragraph 1. The corrected paragraph is shown below.

The 122 participants were a convenience sample of all enrollees in a writing course (described below). They were English majors (111 women; 11 men) who were third year undergraduate students (mean age of 21) at a private university in northeastern China. All spoke Mandarin as their first language and had received formal English training for more than 8 years at the time of the study. However, their average score on the Test for English Majors-Band 4 (TEM-4) was only 50 out of 100, which is a relatively low score. They had not previously received training on peer feedback before the study. Twenty-eight participants were excluded because they failed to submit papers or questionnaires, leaving 94 in the study.

The authors apologize for these errors and state that they do not change the scientific conclusions of the article in any way. The original article has been updated.

## Publisher's Note

All claims expressed in this article are solely those of the authors and do not necessarily represent those of their affiliated organizations, or those of the publisher, the editors and the reviewers. Any product that may be evaluated in this article, or claim that may be made by its manufacturer, is not guaranteed or endorsed by the publisher.
